# The Homeless Poor

**Published:** 1904-03-26

**Authors:** 


### The Hospital.

A Journal of

The Medical Sciences and Hospital Administration.

Vol. XXXV.—No. 913. MARCH 26, 1904.

### The Homeless Poor.

The announcement recently made on the authority of Mr. Shirley Murphy, the Medical Officer of Health for the County of London, to the effect that, on the night of January 29th, the officials acting under his direction counted nearly two thousand homeless persons, men, women, and children, who sought shelter in staircases, under arches, and in all other conceivable places, as soon as those who could not pay for beds were turned out of the common lodging-houses at half an hour past midnight, has not unnaturally appealed very strongly to the charitable, and has led to the suggestion of a variety of schemes for the suppression of what is undoubtedly, and in any possible aspect of the case, a serious, and probably an increasing evil. It is also one which appeals specially to the members of the medical profession as being well-known to supply, and to keep in activity, many sources of possible contagion, both physical and moral; for, apart from the innumerable possibilities of disease which it suggests, there can be no question that some, who in their first experience of homelessness would naturally seek the shelters provided by the law, would be induced by the example of companions in poverty, if not in misfortune, to join themselves to the great body of nocturnal vagrants, and thus to enter upon a downward course, from which return, even if not impossible, would at least be extremely difficult. If our ancestors, in the spacious times of good Queen Bess, erred towards their vagrants in the direction of over severity, and were too liberal of the lash and the pillory, we think it must be conceded that we have gone to the other extreme of the scale, and have displayed an indulgence not only bordering upon weakness, but eminently calculated to increase the evil with which we have to deal. The homeless tramp is a creature who does not possess a single merit as a citizen, and his offspring, unlike those of the Roman *proletarii*, are not in the least degree likely ever to serve their country to the extent of fighting in its defence. Our neighbours in Germany, who are not unduly burdened with sympathy for the undeserving, have already been compelled to seek a practical solution of a problem similar to, but of less extent than, that by which we are confronted; and there are many reasons for thinking that we might properly use their experience as a guide for our own conduct. It is at least certain that the state of things revealed by Mr. Murphy's inquiries is one which ought not to be suffered to continue; and that the interests of public health, as well as of public morality and decency, are alike concerned in the removal of such a blot from our civilisation.

It is not an extravagant assumption to say that every adult person, in exchange for or in acknowledgment of the benefits received from membership of an organised community, owes to that community the duty of bearing his or her proper share of its burdens, and is justly thought guilty of an offence if this duty is not fulfilled. On this view of the case, the utterly unproductive tramp is, *prima facie*, a criminal; and is properly charged with the responsibility of showing that his unproductiveness is excusable. He should be able to establish at least some probability of innocence before his claims to sympathy and to assistance are even considered. Such claims, in many cases, there may doubtless be; and it would be the part of wisdom, as well as of true charity, that they should be taken into account; but the first aspect of the homeless man or woman is that of a person who has failed in the duty of self support, and who is an offender against moral and social laws, even if not against those which are written in the statute book. The first step towards his reform, or towards hindering others from following in his footsteps, would be to bring the statutes into harmony with morality, and to treat the homeless as offenders, but as persons who might possibly be able to urge extenuating circumstances in mitigation of judgment. The proper sequel of such a search as that which Mr. Shirley Murphy conducted would be that the police should be authorised to repeat it, and to take the homeless into custody. The magistrates before whom the offenders were brought should have power to order a period of provisional detention, such as to admit of inquiry into the merits of individual cases, and to sift out those who might be enabled, by judicious charity, to re-establish themselves, from those who were vagabonds only from hatred of work. For the latter, a long period of detention with compulsory labour, with strict separation of the sexes, and with a scale of comforts and indulgences graduated by industry, would be the only proper or possible reformatory treatment. For everything beyond the absolute necessities of life, the apostolic injunction that "if any would not work, neither should he eat," should be carried into rigorous application; and the incurably lazy and useless should be liable to permanent restraint in a settlement so organised as to minimise their opportunities for doing mischief either by act or by example. The cost of the necessary establishment would, of course, be considerable; but against this it must be remembered that the people concerned would, in any case, be maintained at the expense of the public, either by robbery or by foolish almsgiving, and that a penal colony would maintain them in the cheapest possible manner, and would in every way serve to minimise the evils incidental to their existence. Among these, as we have said, and as has been shown by many recent outbreaks of small-pox, the unchecked propagation of infectious diseases holds no inconsiderable place; and on this ground, even if upon no other, the influence of the medical profession may reasonably be exerted in the direction of guiding public opinion towards the adoption of the only methods by which a well-nigh intolerable nuisance can be restrained within limits which ought to be insisted upon by all industrious and respectable members of the community.

